# Monitoring biofilm growth and dispersal in real-time with impedance biosensors

**DOI:** 10.1093/jimb/kuad022

**Published:** 2023-08-31

**Authors:** Matthew McGlennen, Markus Dieser, Christine M Foreman, Stephan Warnat

**Affiliations:** Center for Biofilm Engineering, Montana State University, Bozeman, MT 59717, USA; Mechanical and Industrial Engineering, Montana State University, Bozeman, MT 59717, USA; Center for Biofilm Engineering, Montana State University, Bozeman, MT 59717, USA; Chemical and Biological Engineering, Montana State University, Bozeman, MT 59717, USA; Center for Biofilm Engineering, Montana State University, Bozeman, MT 59717, USA; Chemical and Biological Engineering, Montana State University, Bozeman, MT 59717, USA; Center for Biofilm Engineering, Montana State University, Bozeman, MT 59717, USA; Mechanical and Industrial Engineering, Montana State University, Bozeman, MT 59717, USA

**Keywords:** Biofilm, Quorum-sensing inhibition, Biocide, Electrochemical impedance spectroscopy, Biosensor, Metalworking fluid

## Abstract

Microbial biofilm contamination is a widespread problem that requires precise and prompt detection techniques to effectively control its growth. Microfabricated electrochemical impedance spectroscopy (EIS) biosensors offer promise as a tool for early biofilm detection and monitoring of elimination. This study utilized a custom flow cell system with integrated sensors to make real-time impedance measurements of biofilm growth under flow conditions, which were correlated with confocal laser scanning microscopy (CLSM) imaging. Biofilm growth on EIS biosensors in basic aqueous growth media (tryptic soy broth, TSB) and an oil–water emulsion (metalworking fluid, MWF) attenuated in a sigmoidal decay pattern, which lead to an ∼22–25% decrease in impedance after 24 Hrs. Subsequent treatment of established biofilms increased the impedance by ∼14% and ∼41% in TSB and MWF, respectively. In the presence of furanone C-30, a quorum-sensing inhibitor (QSI), impedance remained unchanged from the initial time point for 18 Hrs in TSB and 72 Hrs in MWF. Biofilm changes enumerated from CLSM imaging corroborated impedance measurements, with treatment significantly reducing biofilm. Overall, these results support the application of microfabricated EIS biosensors for evaluating the growth and dispersal of biofilm *in situ* and demonstrate potential for use in industrial settings.

**One-Sentence Summary:**

This study demonstrates the use of microfabricated electrochemical impedance spectroscopy (EIS) biosensors for real-time monitoring and treatment evaluation of biofilm growth, offering valuable insights for biofilm control in industrial settings.

## Graphical Abstract

**Graphical Abstract:**
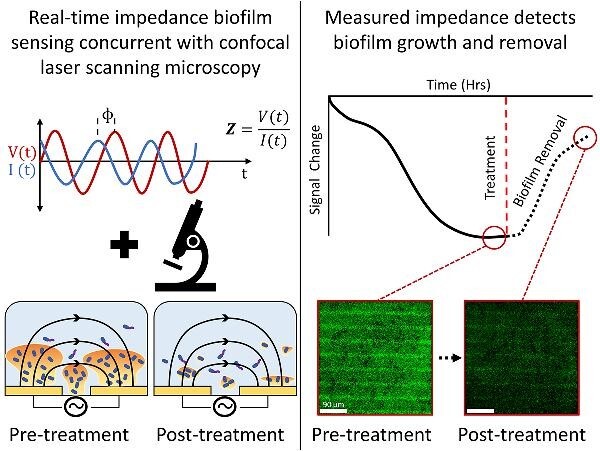
The accurate real-time detection of biofilm growth and removal is effectively demonstrated through the combination of impedance biosensors and high-resolution confocal laser scanning microscopy.

## Introduction

Biofilms are complex, structured microbial communities encased in a self-produced extracellular polymeric substance (EPS) adhered to surfaces. The formation of biofilms is the most common mode of growth for microorganisms on Earth (Costerton et al., 1999) and provides protection from chemical, mechanical, and physical stressors, improves evasion of host defense mechanisms, and increases virulence factors (Lappin‐Scott & Costerton, 1989; Azeredo et al., 2017). Globally, microbial biofilm contamination is a problem in natural, industrial, and medical settings, costing roughly ${\$}$4 trillion annually (Cámara et al., 2022).

Current standard techniques to assess/identify biofilm growth vary in analysis time, sensitivity, cost, and complexity. Standard wet lab techniques include plate counts, dip slides, polymerase chain reactions (PCR), enzyme-linked immunosorbent assays (ELISA), fluorescent *in-situ* hybridization (FISH), and spectroscopic methods such as infrared, ultraviolet, and visible light spectroscopy (Saha & Donofrio, 2012; Saha et al., 2012; Assenhaimer et al., 2017; Kiefer et al., 2018; Passman & Küenzi, 2020). Microsensors have been designed to detect biofilm by changes in oxygen, pH, and temperature (Funari & Shen, 2022; Saccomano et al., 2021) to name a few. Each technique shows both strengths and weaknesses based on its application, highlighting the lack of a one-size fits all approach. Moreover, many of these techniques are destructive endpoint diagnostic tools and cannot be easily deployed to assess in real-time the onset of biofilm formation up to maturation and treatment response.

Over the last decade, microfabricated electrochemical impedance spectroscopy (EIS) biosensors have expanded to the field of microbiology. Compared to standard microbiology techniques, EIS biosensors can obtain information about microbes both in planktonic (Mallén-Alberdi et al., 2016; Brosel-Oliu et al., 2018; McGlennen et al., 2020; Sidhu et al., 2020) and biofilm stages (Chabowski et al., 2017; Huiszoon et al., 2021; Liu et al., 2018). In general, EIS is a technique used to characterize electrochemical properties of solids, liquids, and gases, and is achieved by supplying a sinusoidal electrical perturbation to an electrochemical system and measuring the time-varying response. The measurement is carried out across a frequency sweep (typically ∼1 MHz–1 mHz) (Magar et al., 2021). When applied to biosensors, changes to EIS spectra over time can indicate the presence of microbes at or near the sensor surface. Furthermore, impedance measurements can be carried out at single frequencies, which reduce data acquisition time, simplify analysis, and improve efficiency. Most recently, McGlennen et al. showed that microfabricated EIS sensors using optimized single-frequency impedance measurements could differentiate stages of biofilm growth, starting with the initial attachment of cells through the maturation of biofilm (McGlennen et al., 2023). Based on sensor characteristics and affinity, the analytical detection limit of microfabricated EIS sensor measurements can be less than 10 colony-forming units (CFUs) mL^−1^ (Abdelrasoul et al., 2020). Moreover, EIS sensors are noninvasive and label free, making them desirable for industrial applications and could be expanded into the analysis of formulated liquids.

Biofilm formation is a complex biological process, beginning with initial attachment of cells, proliferation, maturation, and subsequent detachment. Biofilm management has been approached by targeting both the formation process of biofilms and their eradication. Focus has been placed on various additives that interfere with bacterial signaling pathways [i.e., quorum sensing (QS)] or disrupt extracellular DNA, proteins, lipopolysaccharides, and exopolysaccharides during early stages of biofilm formation (Lu et al., 2019; Ozcan et al., 2019). Alternative strategies have also been explored, including the development of antiadhesion coatings, material modifications (Lisoń et al., 2022; Rather et al., 2021), or incorporation of mechanical removal procedures (Sadekuzzaman et al., 2015). Nonetheless, the use of antimicrobials largely remains the standard approach to prevent and remove biofilm (Bott, 2009; Di Pippo et al., 2018). Despite extensive efforts to remove biofilms, microbial contaminants persist, leading to rapid recolonization (Davies, 2003; Stoodley et al., 2004; Stewart et al., 2019; Trafny, 2013). Mature biofilms consist of cells securely attached to the substrate, encased by protective EPS making them resistant to chemical and mechanical forces (Lebeaux et al., 2014; Peterson et al., 2015). Compared to mature biofilms, early-stage biofilms are less securely established, less resistant to antimicrobials, and more susceptible to other chemical treatments (Fu et al., 2021; Høiby et al., 2001). Thus, it is postulated that early detection and treatment could be a highly effective strategy for combating biofilm.

The potential of EIS biosensors to detect early-stage biofilm formation makes them a particularly attractive tool for combatting biofilms. Herein, EIS biosensors were deployed to monitor treatment responses using commercially available anti-biofilm compounds that target a range of different control strategies [e.g., chlorine, biocide, and quorum-sensing inhibitor (QSI)]. Furthermore, the EIS biosensors were tested against biofilms grown in metalworking fluid (MWF), a mixture of oils, emulsifiers, and other additives, to extend their utility beyond simple aqueous environments, as biofilm contamination poses a significant problem in industrial settings (Passman & Küenzi, 2020).

## Material and Methods

### System Overview

**Fig. 1.**
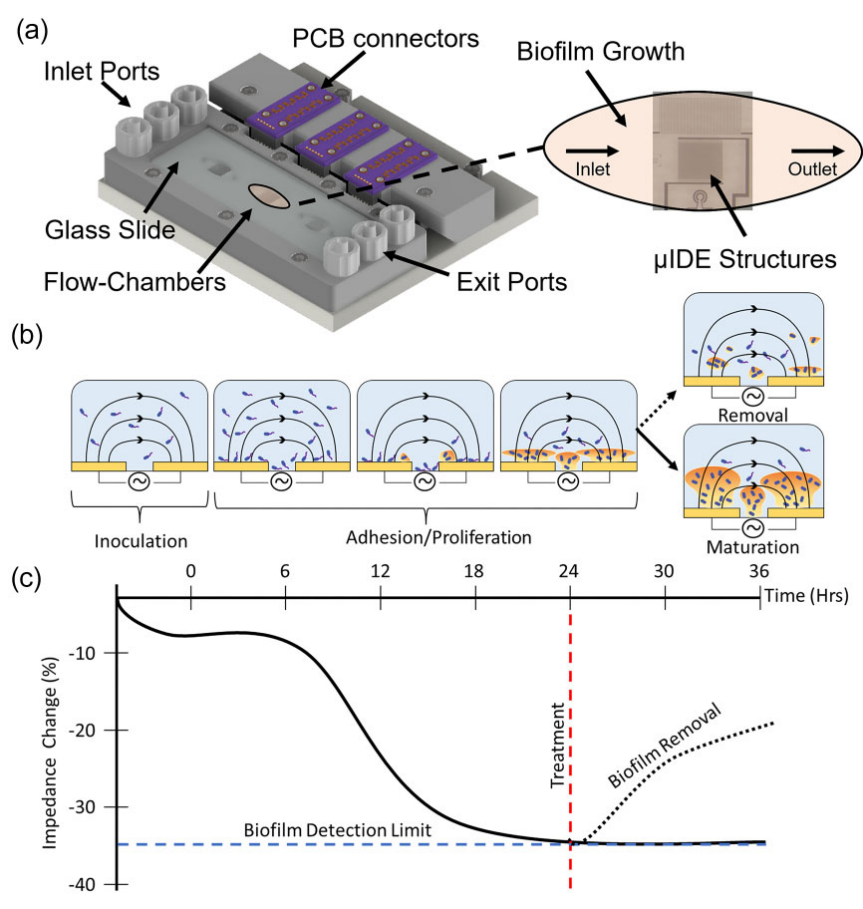
Graphical representation of platform design and experimental setup for analyzing biofilm: (a) 3D printed flow cell system CAD rendering with detailed top view of flow chamber with integrated EIS biosensor for biofilm growth and analysis, (b) cross-section schematic of biofilm development stages on EIS biosensor electrodes within flow chambers, and (c) anticipated impedance changes in response to biofilm growth and removal.

A three-dimensional (3D) printed flow cell system with integrated interdigitated electrode (µIDE) sensors was designed to monitor the growth and treatment of biofilm in a controlled manner, as previously described (McGlennen et al., 2023). The overarching methodology for using the system is depicted graphically (Fig. 1). Briefly, biofilm growth occurs within flow chambers composed of a microfabricated sensor as the bottom substrate, and a glass microscope slide serving as the top substrate viewing chamber (Fig. 1b). Single-frequency impedance analysis indicates the initial attachment of cells to the sensor substrate, early-stage irreversible biofilm proliferation, mature biofilm establishment, and changes due to detachment and regrowth of biofilm (Fig. 1c). Abiotic baseline impedance starts at 0% and decreases correspondingly to increasing biofilm biomass. Impedance changes tend to reach a maximum and stabilize upon uniform biofilm substrate coverage. At this stage, biofilm treatment is introduced, reducing biofilm biomass, and the impedance response correspondingly increases.

### Sensor Design

µIDEs were selected for this application as they have proven to be easily fabricated, are highly sensitive, and do not require the use of a reference electrode (Dorledo de Faria et al., 2019; Furst & Francis, 2019; Park et al., 2018). EIS sensors were microfabricated as previously described (McGlennen et al., 2023). Briefly, the sensor design consisted of interdigitated electrodes 15 µm in width with a spacing of 10 µm for a total of 50 electrode pairs. Sensor surfaces were modified with poly (4-styrenesulfonic acid) doped with pyrrole, termed PPy:PSS, to enhance electrochemical stability and increase sensitivity. One side of each of the µIDEs was coated with 450 µC PPy:PSS before experimentation (McGlennen et al., 2023).

### Flow Cell Operation

Biofilm cultivation was carried out in 3D-printed flow cell systems (McGlennen et al., 2023). Briefly, the flow cell was filled with sterile media (i.e., 1:10X TSB or 5% MWF), and an initial EIS measurement was collected. Subsequently, 1 mL of cell culture enrichments were injected into the flow chambers and left to adhere to the sensor surface for 2 Hrs. Following the 2 Hrs of incubation, a continuous flow of sterile media was initiated into the flow chambers at a rate of 1 µL min^−1^ (Genie Touch Syringe Pump, Kent Scientific, USA). Abiotic (no biofilm) control trials were used to determine the effect of treatment amendments on impedance following the same procedure.

### Bacterial Strain Selection and Culturing Conditions

The model biofilm-forming bacterial strain *Pseudomonas aeruginosa* PA01 expressing green fluorescent protein (GFP) was used (Nivens et al., 2001). Freezer stocks were transferred to tryptic soy agar (TSA; Fischer Scientific, USA) plates and incubated at 37⁰C for 24 Hrs. All cell cultures were grown in the presence of 50 µg ml^−1^ of carbenicillin (Fisher Scientific, USA). Enrichments were made from single colonies grown in 1X tryptic soy broth (TSB; Fisher Scientific, USA) at 37⁰C while shaking at 150 RPM for 18 Hrs.

Following 18 Hrs of incubation, enrichments were harvested by centrifugation at 10 000 × *g* for 5 min. The resulting cell pellets were either resuspended in 1:10X TSB or an industrial oil–water emulsion (i.e., SC-506 CF metalworking fluid (MWF), Hangsterfer's, USA). Cells, which were resuspended in 1:10X TSB, were incubated at 22⁰C while shaking at 150 RPM for 6 Hrs to allow cells to adjust to ambient conditions selected for biofilm growth in the flow cell. Enrichments were harvested at an optical density at a wavelength of 600 nm of OD_600_ = 0.2, resulting in a cell concentration of ∼1 × 10^8^ CFUs mL^−1^. For assays in MWF, cells were washed twice in autoclaved municipal tap water before resuspending cell pellets in autoclaved municipal tap water mixed with MWF (final concentration 5% v/v). The cell concentration of the final mixture was ∼1 × 10^8^ CFUs mL^−1^. All enrichments were transferred to 10 mL sterile luer lock syringes (Becton Dickinson, USA), equipped with 20 ½ ga. needles (Fisher Scientific), prior to injection into flow chambers.

### Biofilm Treatment

The effects of three different biofilm treatments were investigated. After 24 Hrs of growth in the flow cell, established biofilms were treated with either chlorine (Sodium hypochlorite solution; 20% w/v, Sigma–Aldrich, USA) or a biocide (Proxel GXL; Lonza Inc., USA). Treatment concentrations were 1.41 mM chlorine and 0.1% biocide (v/v) in 1:10X TSB and 1.0% biocide (v/v) in 5% MWF. The chlorine concentration was chosen as it has been shown to disperse biofilm in a flow-cell-based system with only partial destruction of biomass (Davison et al., 2010). Proxel GXL was applied at concentrations of 4X and 40X above the recommended minimum inhibitory concentration for planktonically growing *P. aeruginosa*, respectively (Lonza Inc., USA). Chlorine solutions were manually injected as a one-time treatment into each of the three flow chambers (Fig. 1b) at a rate of 1 mL min^−1^ for 1 min. Sterile media flow was paused during chlorine injection and resumed thereafter at a flowrate of 1 µL min^−1^ for 12 Hrs. Biofilm was in contact with chlorine for ∼1.5 Hrs. Conversely, the biocide Proxel GXL was applied as a continuous treatment, in accordance with the manufacturer's recommendations. Sterile media were exchanged with media containing biocide and applied at a flowrate of 1 µL min^−1^ for 12 Hrs.

As a third treatment, biofilm formation was suppressed by targeting QS, a communication process between bacterial cells that regulates the growth and behavior of the microbial population (Miller & Bassler, 2001). Biofilm formation was investigated both in the presence and absence of the quorum-sensing inhibition (QSI) reagent (Z-)-4-Bromo-5-(bromomethylene)-2(5H)-furanone (Furanone C-30; Sigma–Aldrich, USA). Cells, either resuspended in 1:10X TSB or MWF as described above, were supplemented with 75 µM Furanone C-30, as this concentration showed high efficiency at reducing biofilm formation (Ozcan et al., 2019). Enrichments were incubated for 60 min at 22⁰C while shaking at 150 RPM to allow time for the QSI inhibitor to block cell communication prior to flow chamber injection. After inoculating the flow chambers, 75 µM Furanone C-30 mixed with 1:10X TSB or 5% MWF was continuously supplied at a flowrate of 1 µL min^−1^ for either 36 Hrs for 1:10X TSB or 72 Hrs for 5% MWF.

### Electrochemical Impedance Spectroscopy Measurement

EIS measurements were carried out with a Hioki IM3533 LCR (inductance (L), capacitance (C), and resistance (R)) meter connected to a Keysight 970A data acquisition system (DAQ) with a DAQ905A RF 2 GHz Dual 1:4 RF Multiplexer, 50 ohm switching module. The instruments were controlled by a custom-built virtual interface (VI; LabView 2017), providing multiplexed four-channel EIS measurements. During EIS data collection, PPy:PSS-coated sensors were connected to the LCR meter such that the coated side of the µIDEs served as both the counter electrode (CE) and reference electrode (RE). Impedance spectra were collected every 30 min across a frequency range of 200 kHz–100 Hz and 25 logarithmically spaced data points with a signal source that was a sine wave with an amplitude of 10 mV RMS and no DC bias for both 1:10X TSB and 5% MWF. A previous study showed that biofilm detection was most sensitive using 1.26 kHz as the source frequency for impedance measurements in 1:10X TSB (McGlennen et al., 2023). The single frequency that best captured biofilm growth in 5% MWF was 200 kHz (see Supplemental Material). In both cases, time-dependent changes to impedance were reported as given by Equation (1):


(1) \begin{eqnarray*}{\mathrm{Impedance\ Change\ }}\left( {\mathrm{\% }} \right) = \frac{{|{Z}_{\left( {f,t} \right)}\left| - \right|{Z}_{\left( {f,t0} \right)}|}}{{|{Z}_{\left( {f,t0} \right)}|}}\ {\mathrm{*}}\ 100{\mathrm{\% }},\end{eqnarray*}

where |*Z*_(_*_f, t0_*_)_| is the impedance measurement at the selected frequency of interest (*f*), measured at the initial timepoint (*t_0_*), and |*Z*_(_*_f, t_*_)_| is every subsequent impedance measurement.

### Biofilm Assessment with Confocal Laser Scanning Microscopy and Image Analysis

Biofilm experiments and EIS data collection were performed while the flow cell was mounted on a confocal laser scanning microscope (CLSM; SP5 upright confocal, Leica, USA). GFP-expressing *P. aeruginosa* PAO1 was used for all biofilm-related experiments, allowing visualization under the microscope without the need of staining (Nivens et al., 2001). Total biovolume from each CLSM image stack was calculated using Imaris 9.8 surfaces analysis with a custom fluorescence intensity filter and by eliminating particle sizes smaller than 2 µm. The resulting calculation represents the total biovolume present in each confocal stack. The data presented here were normalized to the field of view by using Equation (2), similarly to Lim et al. (2016), creating a biofilm index.


(2) \begin{eqnarray*}{\mathrm{Biofilm\ Index}} = \frac{{\sum {\mathrm{Total\ Biovolume\ }}\left( {\mu {\rm m}^3} \right)}}{{{\mathrm{Field\ of\ View\ }}\left( {\mu {\rm m}^2} \right)}}.\end{eqnarray*}

### Statistical Analyses

Data were processed with Matlab 2021a. Significant impedance changes and biofilm index differences were analyzed using an unpaired *t*-test. Student's *t*-test determined statistical differences between EIS data.

## Results

### Real-time Impedance Measurements of Biofilm Growth

**Fig. 2.**
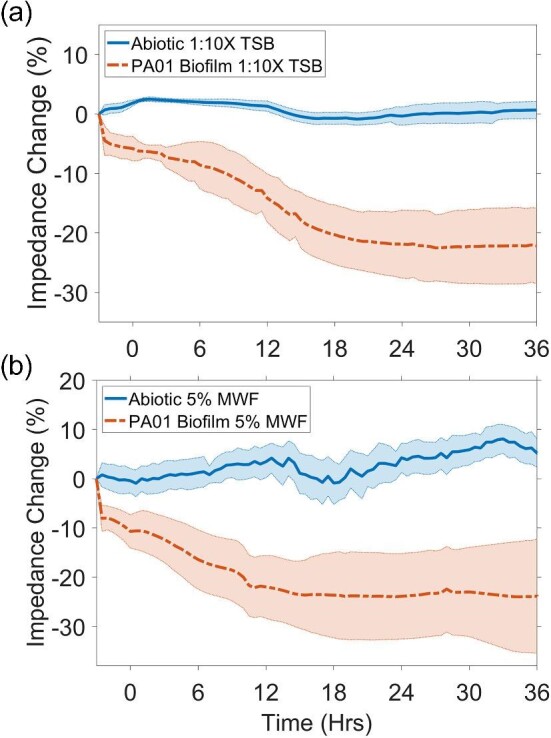
Impedance changes for abiotic controls and biofilm growth in (a) 1:10X TSB and (b) 5% MWF. Shaded regions represent standard deviation.

The flow cell allowed for nondestructive, real-time visualization of biofilm growth and removal without disruption in flow and EIS measurements (Fig. 1). To establish a baseline understanding of the effect of media and the absence/presence of microbial cells, impedance was measured at 1.26 and 200 kHz in 1:10X TSB and 5% MWF, respectively, every 30 min for 36 Hrs (Fig. 2).

Under continuous flow of abiotic 1:10X TSB, impedance remained stable (Fig. 2a, ≤4% variation) and displayed no statistical difference between 0 and 36 Hrs (*t*-Test, *p*-value ≥ 0.079). Injection of planktonic cells into the flow chambers resulted in an instantaneous decrease of ∼5% in impedance. When flow was initiated after 2 Hrs of seeding (i.e., 0 Hr) impedance decreased in a sigmoidal decay pattern until reaching a plateau at ∼−22% by 24 Hrs of growth (24–36 Hrs, *t*-Test, *p*-value = 0.956). Visual confirmation of the different biofilm stages from seeding to monolayer establishment in relation to EIS can be found in McGlennen et al. (2023).

Similar to impedance changes in 1:10X TSB, impedance displayed no statistical difference between 0 and 36 Hrs of continuous flow under abiotic conditions in 5% MWF (*t*-Test, *p*-value ≥ 0.051). In the presence of cells, impedance attenuated in a sigmoidal decay pattern, reaching a plateau of ∼−25% by 24 Hrs of growth (24–36 Hrs, *t*-Test, *p*-value = 0.996).

### Real-time Impedance Measurements of Biofilm Treatment

**Fig. 3.**
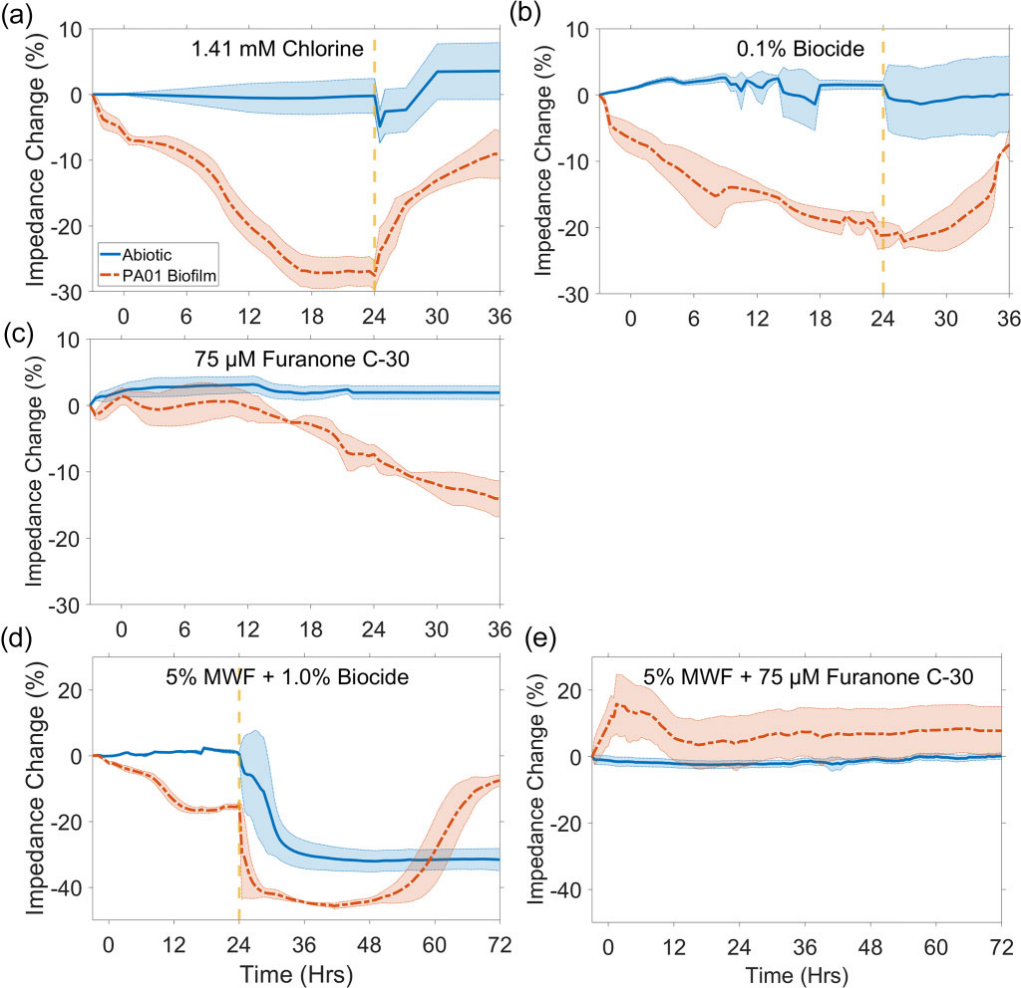
Impedance changes at 1.26 kHz over time for abiotic controls and biofilm grown in 1:10X TSB: (a) treated at 24 Hrs with 1.41 mM chlorine, (b) treated at 24 Hrs with 0.1% biocide, (c) exposed to 75 µM furanone C-30 for 36 Hrs. Impedance changes at 200 kHz over time for abiotic controls and biofilm grown in 5% MWF, (d) treated at 72 Hrs 1.0% biocide, and (e) exposed to 75 µM furanone C-30 for 72 Hrs. Shaded regions represent standard deviation. Dashed line indicates time of treatment implementation.

**Fig. 4.**
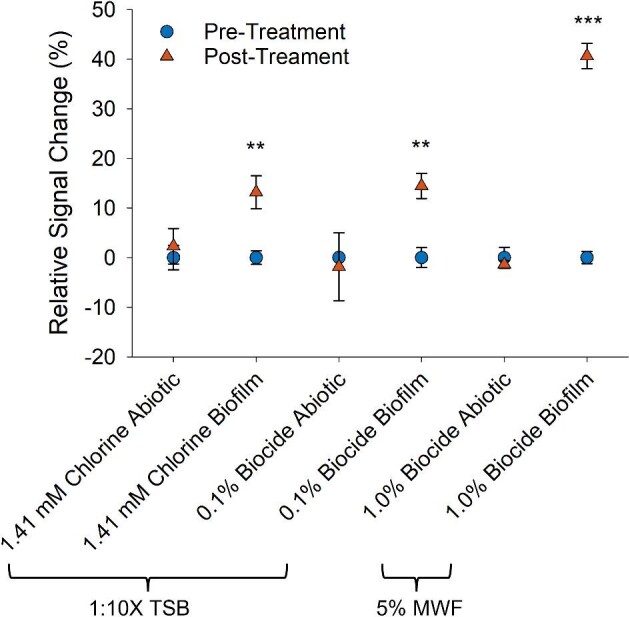
Magnitude of impedance change between pre-treated and post-treated biofilm grown in 1:10X TSB and 5% MWF. Error bars indicate standard deviation. Differences that met significant levels are shown as *≤0.05, **≤0.01, ***≤0.001.

To investigate biofilm removal using EIS biosensors, 24-Hr biofilms were treated with either 1.41 mM chlorine or 0.1% biocide. The addition of these agents altered the electrochemical properties of 1:10X TSB and 5% MWF, and hence, the impedance baseline. Effects on impedance were noisier in the abiotic media compared to biosensors overgrown with biofilm, which increased impedance variability by 10 and 7% on average in abiotic 1:10X TSB and 5% MWF, respectively (Fig. 3a,b). The addition of chlorine decreased impedance sharply by ∼5% in abiotic 1:10X TSB and plateaued 6 Hrs after treatment with an overall impedance increase of ∼5% (Fig. 3a). Impedance changes caused by the addition of chlorine and biocide to 1:10X TSB were, however, not statistically significant (*t*-test, *p*-value ≥ 0.414; Fig. 4). In 5% MWF, the addition of the biocide triggered a large drop in impedance of ∼30% before stabilizing 12 Hrs later for both abiotic sensors and sensors exposed to biofilm (Fig. 3d). Impedance remained stable between 36 and 72 Hrs in abiotic 5% MWF (*t*-Test, *p*-value = 0.339; Fig. 4); thus, the impedance at 36 Hrs was selected as the new baseline for treatment effect calculations.

All biosensors covered with biofilm reflected a treatment response upon contact with chlorine and biocide, with impedance changes nearing abiotic baselines (Fig. 3). Differences were evident with respect to response time. Impedance changes for biosensors treated with a one-time injection of chlorine were instant and followed a saturation curve pattern (Fig. 3a). During continuous treatment of biofilm in both 1:10X TSB and 5% MWF with a biocide, impedance changes followed a sigmoidal pattern with a pronounced lag phase (Fig. 3b,d). Overall, impedance increased by 13 ± 3% and 14 ± 3% for chlorine and biocide-treated biofilms in 1:10X TSB, respectively, and by 41 ± 3% for biocide-treated biofilms in 5% MWF. Importantly, changes in impedance were statistically significantly different between key timepoints of pre-treatment (24 Hrs) and post-treatment (36 Hrs) for EIS biosensors treated with chlorine (*t*-Test, *p*-value = 0.003) and biocide (*t*-Test, *p*-value = 0.002) in 1:10X TSB (Fig. 4). Likewise, in response to biofilm treated with biocide in MWF, the impedance change was statistically different between pre-treatment (36 Hrs) and post-treatment (72 Hrs) (*t*-Test, *p*-value < 0.001).

### Real-time Impedance Measurements of Delayed Biofilm Development

To investigate whether EIS biosensors could monitor the effects of QSI on biofilm formation, cell enrichments were grown in the presence of 75 µM of the QSI compound furanone C-30 for 36 and 72 Hrs in 1:10X TSB and 5% MWF, respectively. Electrochemically, the addition of furanone C-30 had negligible effects on impedance (Fig. 3c,e). Compared to 0 Hr, impedance remained stable in abiotic 1:10X TSB (*t*-test, *p*-value = 0.811) and abiotic MWF (*t*-test, *p*-value = 0.246) for 36 and 72 Hrs, respectively. Impedance indicated that biofilm proliferation was suppressed in 1:10X TSB and 5% MWF for 18 and 72 Hrs, respectively (*t*-test, *p*-value ≥ 0.329; Fig. 3c and e). In 1:10X TSB, changes in impedance became statistically different from 0 Hr after 18 Hrs (*t*-test, *p*-value = 0.016). A gradual decrease in impedance, reaching ∼−17% by 36 Hrs, suggested that cells eventually overcame the inhibitory effects of furanone C-30 and biofilm began to develop.

### Validation of Biofilm Removal and Delay By CLSM Imaging

**Fig. 5.**
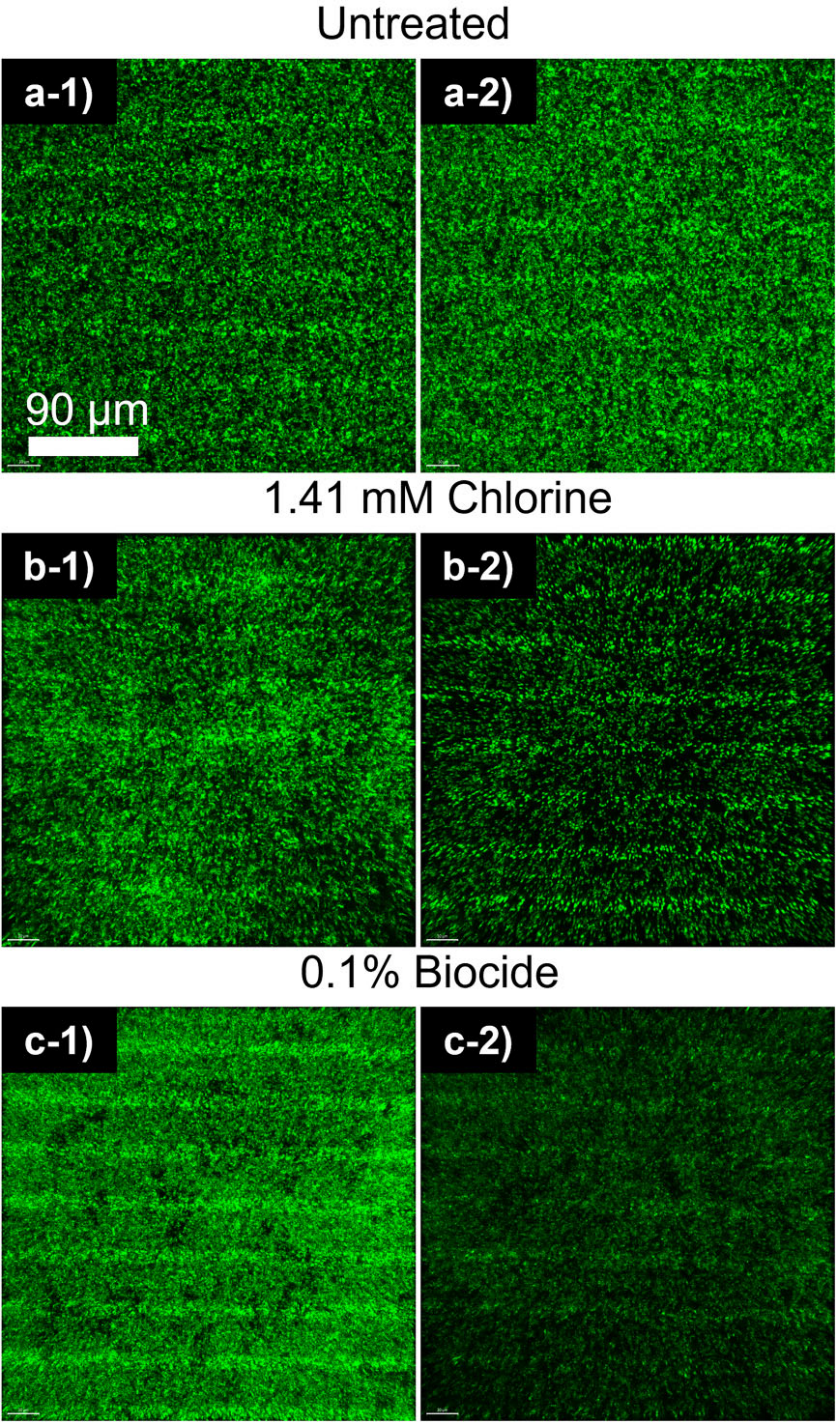
CLSM images of (left panel: −1) pre-treatment (24 Hrs) and (right panel: −2) post-treatment (36 Hrs) biofilm. (a) Untreated, (b) chlorine treatment, and (c) biocide treatment. Scalebar represents 90 µm.

**Fig. 6.**
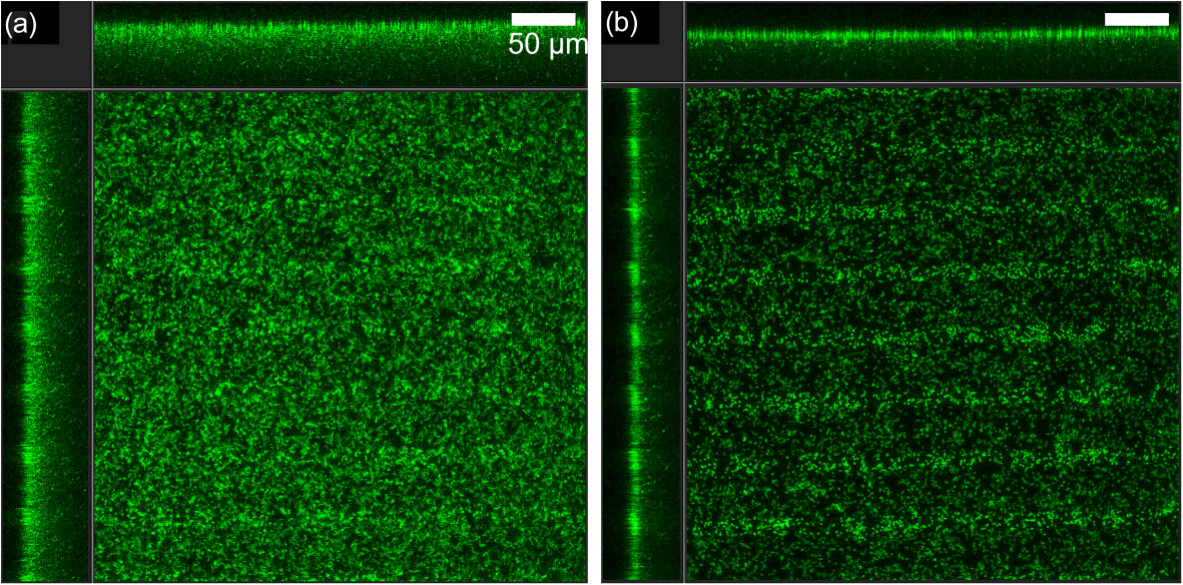
CLSM images of (a) untreated biofilm at 36 Hrs, and (b) biofilm exposed for 36 Hrs to 75 µM of the QSI compound Furanone C-30. Scalebar represents 50 µm. Top and side panels show side-view profiles of biofilm z-stacks.

CLSM imaging was used to confirm biofilm development and treatment in 1:10X TSB media but was not possible in 5% MWF as the fluid is opaque. CLSM images at key timepoints of pre-treatment (24 Hrs) and post-treatment (36 Hrs) were evaluated (Fig. 5). Consistently, biofilm monolayers attached to biosensor substrata had formed after 24 Hrs (Fig. 5a-1, b-1, and c-1) and continued to proliferate without treatment (Figs. 5a-2). Conversely, exposure to either chlorine or biocide removed biofilm (Figs. 5b-2 and c-2). Biofilm removal was quantified using Imaris 9.8 by calculating changes in biofilm index (B.I.; Equation 2) between pre-treatment and post-treatment. Chlorine and biocide treatment resulted in an average B.I. reduction of 1.87 ± 0.75 µm^3^ µm^−2^ and 2.13 ± 0.89 µm^3^ µm^−2^ between pre-treatment and post-treatment, respectively. Both treatments resulted in a significant reduction of biofilm when compared to biofilm without treatment (*t*-Test, *p*-value ≤ 0.024). Cells treated with 75 µM furanone C-30 developed thinner biofilms, exhibiting ∼40 % reduction in overall thickness compared to untreated biofilms (Fig. 6).

## Discussion

The developed platform consisted of EIS biosensors integrated into a microfluidic flow environment, allowing for impedance measurements that reflect naturally proliferating biofilm conditions. Live-cell imaging from CLSM supplemented impedance data as a nondestructive visual confirmation of adherent biofilm. The marriage of these characteristics makes the current report unique in that biofilm growth under flow conditions and the expansion of the sensors into alternative fluids and QSI is explored. Extending findings by McGlennen et al. (2023) on linking biofilm growth to EIS measurements, this study assessed the applicability of impedance to monitor biofilm treatment and the application of QSI in real-time. The behavior of microfabricated sensors used to evaluate biofilm growth is highly dependent on the experimental setup, such as the type of media, flow characteristics (e.g., static vs. continuous flow), organism selection, electrode geometry (e.g., 2- vs. 3-dimensional), and selected frequency range (Furst & Francis, 2019; Subramanian et al., 2020; Funari & Shen, 2022). All of these factors complicate direct comparisons between reports.

In the present study, a flow-based system using µIDEs coated with an electrically conductive polymer (i.e., PPy:PSS), measured at an optimized single frequency (i.e., 1.26 and 200 kHz) was deployed. Impedance decreased in the presence of proliferating biofilm following sigmoidal decay curves (Figs. 2 and 3). Others have found a similar relationship between biofilm growth and impedance (Paredes et al., 2012, 2013, 2014; Subramanian et al., 2017; Huiszoon et al., 2019). These studies reported impedance changes ranging from ∼10 to ∼40%, and plateaus were reached between 10 and 48 Hrs, bracketing our findings of 20–25% before leveling off at ∼24 Hrs. Likewise, impedance in the low-frequency ranges (i.e., 10 Hz–10 kHz) was most representative of signals from the electrode/electrolyte interface and provided the highest sensitivity toward biofilm growth (McGlennen et al., 2023; Paredes et al., 2014). While the frequency range in 1:10X TSB growth medium was optimized at 1.26 kHz, this frequency was not suitable for MWFs. Industrial oil–water emulsions can form rigid dielectric barriers that hinder the mobility of charge carriers at the interface of the electrode (Perini et al., 2012), suggesting that higher frequencies may be more effective in measuring biofilm. We found that the most appropriate frequency range for measuring biofilm in 5% MWF was ≥100 kHz, as detailed in the supplemental information. However, it should be noted that the degree of hindrance to charge carriers can vary depending on the amount of water in the emulsion, and optimal sensor signal responses may need to be tailored to individual systems. MWF was chosen as a representative medium to test EIS biosensors since MWF is prone to *P. aeruginosa* biofilm contamination (Di Maiuta et al., 2017; Passman & Küenzi, 2020). Importantly, EIS detected biofilm growth in MWF with the same degree of sensitivity as in 1:10X TSB (Figs. 2 and 3).

While the exact mechanisms behind the impedance decrease in response to biofilm are not fully understood, we hypothesize that biofilm metabolic processes modify ionic concentrations at the capacitive double-layer of the sensor electrodes. This leads to a decreased Debye length, and thus, an elevated capacitive double-layer that results in a decline in impedance (Huiszoon et al., 2019, 2021; McGlennen et al., 2023). Additionally, upon the production of EPS, electrochemically active metabolites such as phenazine, pyocyanin (PYO), or *Pseudomonas* quinolone signal (PQS) may become entrapped within the EPS matrix, leading to the accumulation of electrochemically active sites (Becerro et al., 2016; Bosire et al., 2016). Cells may also act as small capacitors, decreasing impedance (i.e., capacitance increase; Turick et al., 2019). Conversely, biofilm treatment causes damage to cells and/or disruption of the EPS matrix, and decreases metabolic processes, resulting in the release and subsequent decrease in the concentration of electroactive redox compounds. These compounds are then carried away by the flow, and thus, the impedance increases. With regards to these mechanisms, chlorine causes the detachment of biofilm clusters and the breakdown of metabolites by oxidation of organic materials (Davison et al., 2010), whereas 1,2-benzisothiazolinon-3-one (i.e., biocide) is believed to inhibit bacterial growth and metabolic activity (Green et al., 2018; Silva et al., 2020). Both chlorine and biocide treatments resulted in impedance increases (Figs. 3b and c). While indicative of biofilm reduction, impedance did not fully rise to abiotic levels. Further, the reduced slope of the impedance curves not only implied incomplete removal of biofilm but also that biofilm became less susceptible to the treatment. Incomplete biofilm removal was confirmed by CLSM (Fig. 5). The measured ∼14% impedance increase corresponded to an ∼68% decrease in biomass. However, it is important to note that this study aimed to detect changes in impedance in response to biofilm treatment rather than optimizing biofilm treatment doses.

Furanone C-30 is a QSI compound and has been demonstrated to delay biofilm formation and maturation (Wu et al., 2004; He et al., 2012; Zhao et al., 2018). QS is a type of bacterial communication to sense and respond to cell population density, mediated by chemical signaling molecules (Mukherjee & Bassler, 2019). Briefly, the abundance of signaling molecules is low or absent at low cell densities. The production, release, and extracellular concentration of signaling molecules increase as a function of growing cell density, ultimately triggering a synchronized cell population-wide response such as biofilm formation and architecture (Davey et al., 2003). As such, QS systems are autoregulated and depend on a threshold concentration of signaling molecules (Miller & Bassler, 2001). By destabilizing QS regulatory proteins, furanone C-30 decreases the synthesis of signaling molecules [i.e., acyl-homoserine lactones (AHLs) and PQS] (Markus et al., 2021; Skindersoe et al., 2008), which further downregulates the production of other redox mediators such as phenazine and PYO (Davies et al., 1998; Hentzer et al., 2003; Proctor et al., 2020). When biofilm was grown in the presence of furanone C-30, impedance remained unchanged for 72 Hrs in 5% MWF (Fig. 3e). In 1:10X TSB, impedance remained stable for 18 Hrs (Fig. 3c), and the subsequent decrease in impedance indicated that QSI was overcome. Ozcan et al. tested *P. aeruginosa* PAO1 biofilm growing in both TSB and MWF in the presence of furanone C-30, and reported lower concentrations of signaling molecules (PQS and AHLs) critical to biofilm development (Ozcan et al., 2019), affirming the potential role of these mediators in affecting impedance.

The use of µIDEs in combination with PPy:PSS electrode coatings results in highly sensitive biofilm detection and enhanced measurement integrity/repeatability (this study; McGlennen et al., 2023). Recent reviews have identified four criteria relevant for developing robust biofilm detection tools: Analytical techniques need to be (*i*) noninvasive (i.e., cannot perturb or damage biofilm), (*ii*) provide real-time analysis with high temporal and spatial resolution, (*iii*) be applicable to diverse analytes and settings, and (*iv*) be cheap, require low maintenance, and operate continuously for a long time (Funari & Shen 2022; Saccomano et al. 2021). The EIS biosensors developed herein hold promise to fulfill these criteria as they have the advantages of being able to be manufactured at low cost and can be configured for automated data collection and interpretation, all of which results in a higher efficiency and real-time evaluation of biofilm. To take advantage of the capabilities of EIS, further research is needed to better understand the mechanisms that drive changes in impedance. For instance, this study investigated the model organism, *P. aeruginosa* PA01, but other organisms likely have different electrochemical compositions. It will be necessary to assess the performance of these sensors in diverse biofilm compositions (e.g., mixed species and/or inter-kingdom), different types of supporting fluids (e.g., opaque, liquid formulations), and under varying environmental conditions (e.g., fluctuations in temperature, conductivity, or pH). If further validated, impedance sensors would be a significant advancement where biofilm contamination is prevalent and the reliance on current monitoring techniques are inadequate.

## Conclusion

In this study, microfabricated EIS biosensors were effective at assessing the growth and removal of *P. aeruginosa* biofilm in 1:10X TSB and 5% MWF. Additionally, EIS biosensors detected delayed effects of biofilm growth from targeting bacterial QS inhibition. The use of EIS biosensors holds great potential for the early detection and continuous monitoring of biofilms. Expanding our understanding of the sensors’ uses and limitation beyond laboratory-based testing is vital to ensure future technological expansion into industrial, medical, and natural environments.

## Supplementary Material

kuad022_Supplemental_Files

## Notes

Imaging was made possible by the Center for Biofilm Engineering Imaging Facility at Montana State University, which is supported by funding from the National Science Foundation MRI Program (2 018 562), the M. J. Murdock Charitable Trust (202 016 116), and the US Department of Defense (77369LSRIP).
