# Supplementary material for: Monitoring biofilm growth and dispersal in real-time with impedance biosensors
Source: J Ind Microbiol Biotechnol. 2023 Aug 31;50(1):kuad022. doi: 10.1093/jimb/kuad022 (PMC10485796; doi:10.1093/jimb/kuad022)
Supplement: kuad022_Supplemental_Files [file kuad022_supplemental_files.zip › Supplemental_information_McGlennen_et_al_2023_manuscrip.pdf]

**Supplementary Information for:**

# Monitoring Biofilm Growth and Dispersal in Real-Time with Impedance Biosensors

Matthew McGlennen,[1,2] Markus Dieser,[1,3] Christine M. Foreman,[1,3] Stephan Warnat[1,2, *]

1 Center for Biofilm Engineering, Montana State University, Bozeman, MT

2 Mechanical and Industrial Engineering, Montana State University, Bozeman, MT

3 Chemical and Biological Engineering, Montana State University, Bozeman, MT

* Corresponding Author:

Stephan Warnat

stephan.warnat@montana.edu

Montana State University

Roberts Hall 304, Bozeman, MT 59717

(406) 994-6284

Compared to TSB, MWF has properties that effected biosensor responses. We postulate that the thick, oily layer created by MWF constituents reduced sensitivity at the electrochemical interface, thus masking measurable interactions between biofilm and the electrodes. To determine the optimal frequency range that best captured biofilm growth in 5% MWF, abiotic and biofilm growth experiments were carried out in the flow cell system. Impedance changes at timepoints t=initial, 0, 6, 12, 18, 24, 30, and 36 hrs under flowing conditions were measured (Supplemental Figure 1). Frequency ranges of interest were defined at low (100≤f≤1 kHz), medium (1 kHz≤f≤10 kHz), or high frequency (10 kHz≤f≤200 kHz). Under abiotic conditions in 5% MWF, impedance changes across the entire frequency range (100 Hz≤f≤200 kHz) fluctuated less than 10% without a trend for 36 hrs (Supplemental Figure 1).

However, in the presence of biofilm, impedance in the high-frequency range was most pronounced (≤25%) and consistently decreased with respect to time (Fig 4b). Unlike TSB, biofilm in 5% MWF impedance in the low and middle frequency ranges fluctuated with no trend (≤10%). The high-frequency range (10 kHz≤f≤200 kHz) was identified as the most suitable frequency range to measure biofilm in 5% MWF due to better sensitivity. Therefore, 200 kHz was chosen as the single representative frequency to measure biofilm in 5% MWF.

**Supplemental Figure 1** a) Relative impedance response of entire measured frequency range at selected timepoints of 0, 6, 12, 18, 24 hrs of 5% MWF flow in flow-cell under abiotic conditions. b) Relative impedance response of entire measured frequency range at selected timepoints of 0, 6, 12, 18, 24 hrs of 5% MWF flow in flow-cell under biofilm growth conditions**.**

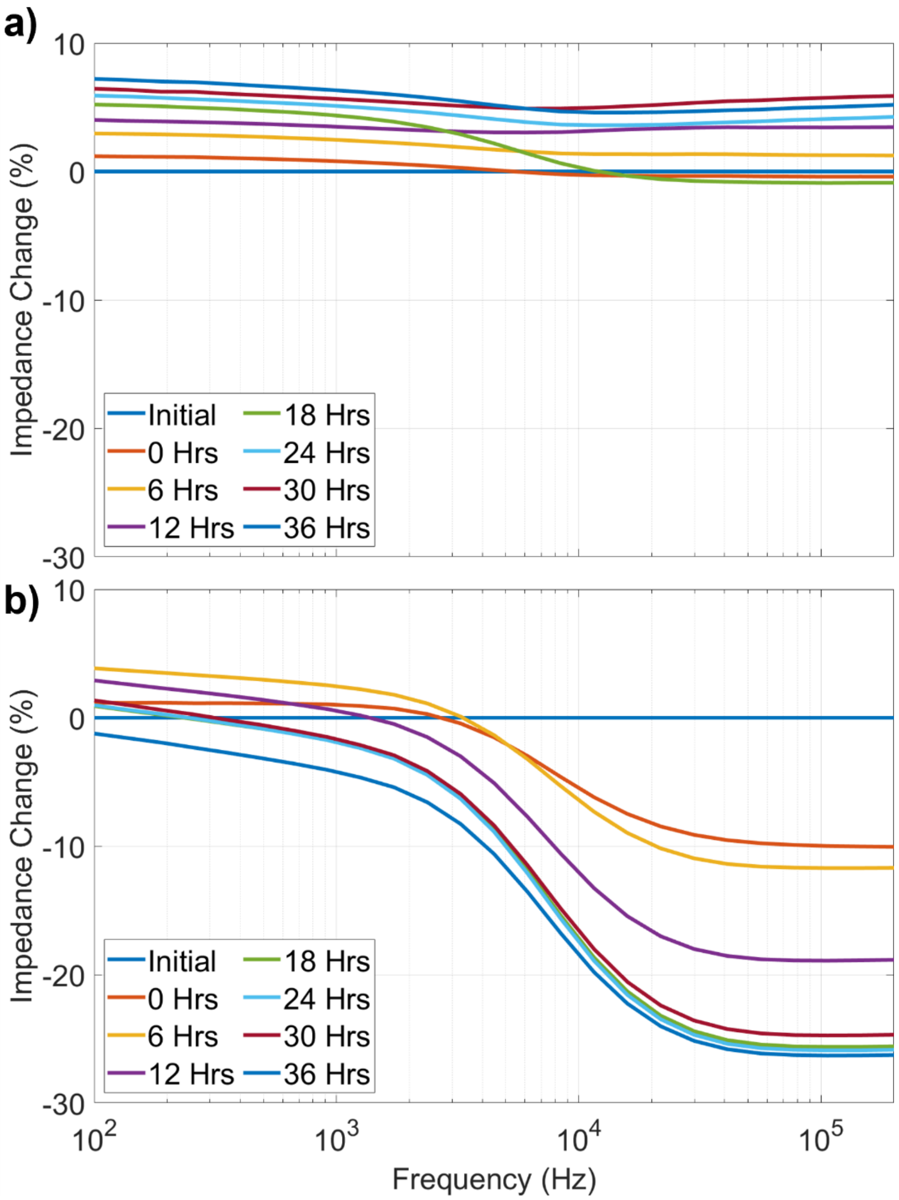


Supplemental Figure 1
