# Supplementary figures and images for: Monitoring biofilm growth and dispersal in real-time with impedance biosensors
Source: J Ind Microbiol Biotechnol. 2023 Aug 31;50(1):kuad022. doi: 10.1093/jimb/kuad022 (PMC10485796; doi:10.1093/jimb/kuad022)

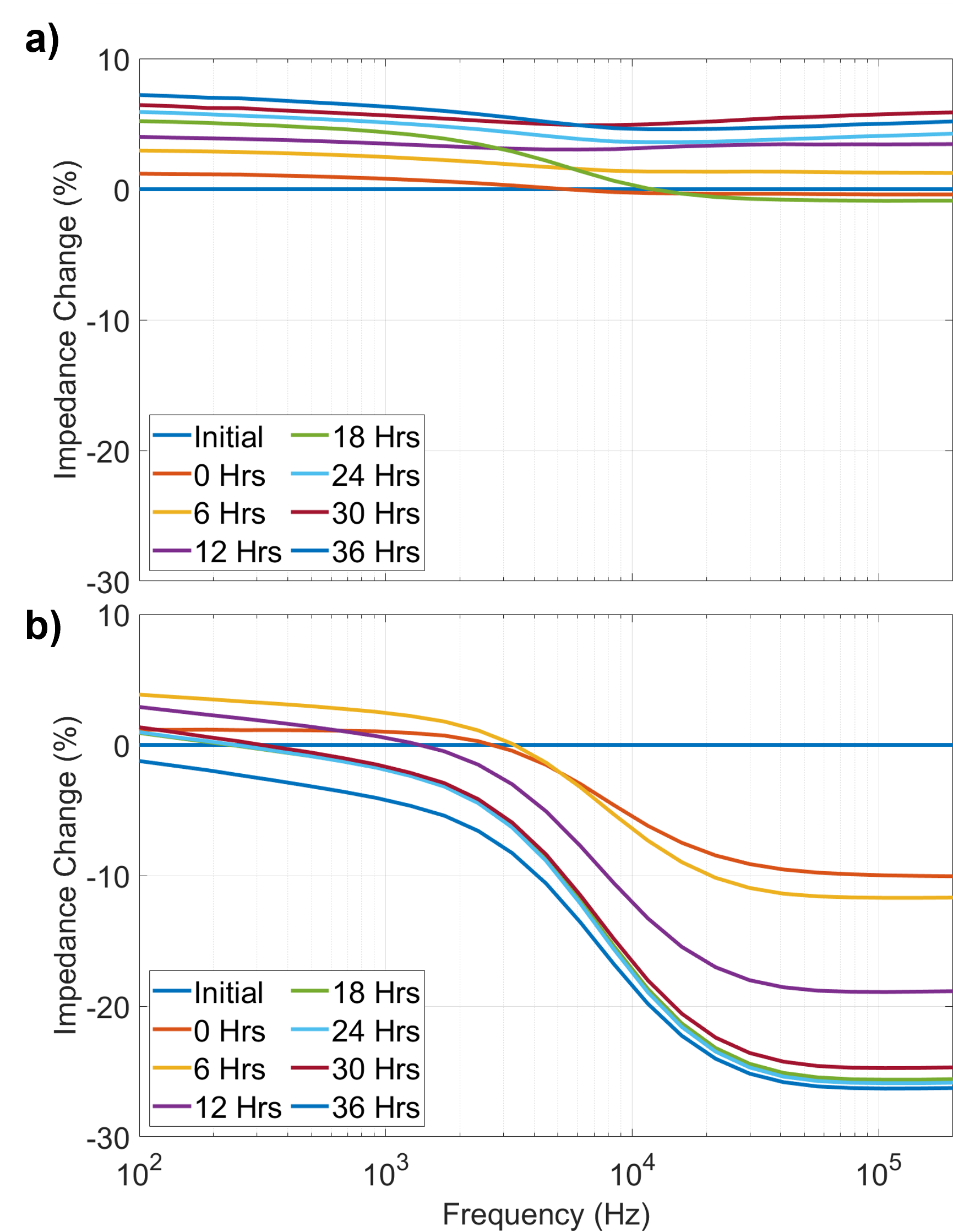

Supplement: kuad022_Supplemental_Files [file kuad022_supplemental_files.zip › Supplemental_Fig1.tif]
